# Dysphagia services in the era of COVID-19: Are speech-language therapists essential?

**DOI:** 10.4102/sajcd.v67i1.709

**Published:** 2020-07-29

**Authors:** Kim A. Coutts

**Affiliations:** 1 Department of Speech Pathology, Faculty of Humanities, University of the Witwatersrand, Johannesburg, South Africa

**Keywords:** dysphagia, COVID-19, essential services, speech language therapy, swallowing

## Abstract

In the era of coronavirus disease 2019 (COVID-19), many healthcare professionals are being faced with the question of what is considered to be an essential service. This opinion paper has attempted to answer this complex question by understanding the potential relationship between dysphagia and COVID-19 and how speech-language therapists (SLTs) in South Africa should tackle this. It also aims to answer the question through the lens of a risk–benefit discussion based around practices and decision-making. Important gaps in the field relating to how SLT practices need to move forward during this challenging time have also been highlighted. Reflective questions that can assist SLTs when seeing dysphagia cases have been provided.

## Introduction

This challenging and somewhat scary era, during which we are facing the threat of the coronavirus disease 2019 (COVID-19) on a daily basis, is constantly evolving. As healthcare workers, we need to respond to this by also evolving in both our thinking and practices. This pandemic is resulting in severely limited resources in all healthcare settings globally, and as a result, the roles and scopes of many professionals are being redefined. This is a complex topic with many aspects that still need to be discussed. There are also a number of critical questions that are being asked, especially amongst rehabilitation professions. This article will attempt to address one question: Should dysphagia services by speech-language therapists (SLTs) be considered essential in this COVID-19 era in the South African (SA) context?

## Dysphagia and COVID-19: A brief overview

COVID-19 is a novel coronavirus that impacts the functioning of the respiratory system (World Health Organization [WHO], 2020). It also appears to be severely affecting the more at-risk populations, who are considered to be the elderly (people over the age of 60 years), people with pre-existing lung conditions as well as individuals with already underlying medical conditions that have resulted in a weakened immune system (WHO, 2020). Because COVID-19 affects the respiratory system primarily, this is significant for speech-language therapists (SLTs), as these are patients who are at risk for also developing a dysphagia. This link will be explored below.

Dysphagia can lead to some significant medical consequences, including but not limited to dehydration, malnutrition, aspiration and aspiration pneumonia (Abu-Ghanem, Chen, & Milan, 2020). These factors alone can lead patients to a longer hospital stay, which has resource implications for the healthcare facility as well as an impact on the patient in terms of their well-being (Martino et al., 2005). The relationship between COVID-19 and dysphagia is still a new area, but there is enough understanding in the field to create links between the two. As the respiratory system is closely coordinated with the normal swallowing pattern, if a patient were to present with significant respiratory complications, the patient could be at risk of developing a dysphagia, which can result in aspiration (Matsuo & Palmer, 2009). If the normal breathing pattern is disrupted, the airway is potentially exposed as a result of this uncoordinated period of apnoea, which may result in inadequate airway closure (Nishino, 2012). This phenomenon can be seen with the high prevalence of dysphagia in individuals with chronic obstructive pulmonary disease (COPD) (Scelza, Greco, Lopes, & De Melo, 2015), for example.

It is also known that patients with COVID-19 often require extended periods of ventilation – for longer than 72 h – and this alone can lead to an increase in post-extubation dysphagia (PED), which is already prevalent (Bhatraju et al., 2020) in these now-overcrowded intensive care units (ICUs). Prevalent post-extubation dysphagia, if unidentified, can lead to further complications such as aspiration and aspiration pneumonia, and these will increase the patient’s length of stay in the ICU as well as lead to a higher mortality rate (Patel et al., 2018; Perren, Zürcher, & Schefold, 2019). Luis Riquelme made a fair assumption when he stated that COVID-19-positive patients with an impaired respiratory system who then present with a PED may not present typically, as this is a new disease (The Role of the SLP during COVID 19 – YouTube, 2020). These new and unknown factors are something that SLTs need to be aware of when assessing and managing these patients. The role of SLTs in identifying and managing these cases is therefore also vital.

There is also evidence emerging that COVID-19 patients can present with neurological deficits (Mannan Baig, Khaleeq, Ali, & Syeda, 2020). The extent of these deficits is still being determined, but it is probably not a far reach to assume that a potential consequence of these neurological fallouts could be dysphagia, depending on where the site of the lesion is. The description of these neurological deficits in COVID-19 patients still requires extensive investigation in all settings. In the SA population, where a vast portion of the population presents with various communicable and non-communicable diseases that impair the immune system, how COVID-19 will manifest is also yet to be fully determined. These COVID-19 manifestations, together with the already known diseases in the SA population, can have a further significant impact on who SLTs may need to treat and for what intervention in the already overcrowded, underserviced public healthcare system.

In terms of dysphagia in COVID-19 patients, the main consideration needs to be that the most severe consequence is an aspiration pneumonia. A patient presenting with COVID-19 who either has or subsequently develops an aspiration pneumonia would then become severely ill, therefore further complicating the respiratory effects of the virus and affecting the overall recovery time. This situation needs to be prevented at all costs, especially in the SA context where access to scarce resources such as ICU beds is a challenge.

## The argument

In an attempt to shed light on the question at hand, we need to examine it from the perspective of a risk–benefit discussion. This simple framework attempts to weigh the clinical benefits of a dysphagia intervention against the possible side effects or implications that can arise from this type of contact. It can also be expected that the answer to this question would be slightly different depending on the context in which the SLT is employed, for example, acute care versus long-term care or a rehabilitation setting.

### The benefits

There are some significant medical complications associated with an untreated dysphagia (Abu-Ghanem et al., 2020), and the goal of early rehabilitation (including the need for an SLT) in an acute setting is to decrease the potential medical complications that can arise from the dysphagia itself or as a consequence of being in these settings, such as PED. It is also to decrease the length of stay of a patient in a healthcare facility (Taito et al., 2018). In the COVID-19 era, when lack of availability of resources such as ICU beds, ventilators and personal protective equipment (PPE) further exacerbates an existing challenge (Naidoo, Singh, & Lalloo, 2013), this practice of early rehabilitation initiation is paramount. This article argues that SLTs need to be in the forefront of this pandemic in order to reduce the complications that can arise from being in an ICU setting together with other rehabilitation professionals (Hu, Hsu, Yip, Jeng, & Wang, 2010).

In the more long-term and rehabilitation settings, the intended outcome of SLT intervention still would be to prevent the serious medical sequelae of dysphagia. This intervention should still be prioritised in these settings, as the main population there are considered to be the most at risk (often elderly people with underlying medical conditions) for developing the serious sequelae of COVID-19. By preventing these medical consequences of dysphagia whilst patients are still in old-age homes or rehabilitation settings, SLTs are preventing these patients from being admitted into hospitals, the epicentres of treatment for COVID-19 patients. By identifying these patients early, these consequences and hospital visits can be avoided. However, this begs the question, does this mean that every person needs a dysphagia screening or assessment by an SLT, considering that we are now in the era of social distancing and PPE scarcity? Perhaps a patient risk profile needs to be developed in order to identify patients in order of priority and severity risk? The new South African Speech-Language-Hearing Association (SASLHA) Dysphagia Guidelines for COVID-19 (2020) appear to also focus on this type of approach of triaging and risk identification. The need for screening and assessing all patients in this era could lead to some significant risks for both patients and SLTs.

### The risks

Let’s start with a broad statement about SLT services generally. All SLT services place the clinician at risk for airborne diseases, as our work involves functions of both the upper and lower respiratory tract – talking, breathing and swallowing. This alone is something that we as SLTs need to be cognisant of. That said, let us focus on dysphagia. This could arguably be the most high-risk area that an SLT could be involved in, specifically in this COVID-19 era. The assessment and management of dysphagia cases would involve aerosolization within close contact. The recent SASLHA Dysphagia Guidelines for COVID-19 patients (2020) state specifically that oral care, cough reflex testing, implementation of videofluoroscopic swallow studies (VFSS) or flexible endoscopic evaluation of swallowing (FEES), interventions with tracheostomy and ventilator dependency and laryngectomy are considered risky procedures. These are potential aerosol-generating procedures (AGPs), and SLTs need to be careful when conducting them.

These close-contact procedures can place both the clinician as well as the patient at risk for transmission of the virus. Aerosol-generating procedures are risky for the clinician, but an SLT seeing multiple patients for these AGPs can place the patient at risk for potential cross-contamination (Rodriguez, 2020). It is best practice at this point to assume that everyone is considered positive, and all necessary precautions should be taken to avoid transmission during sessions and to avoid cross-contamination between patients who are being seen by the SLT. In order to do this, the appropriate PPE needs to be used in all cases (Rodriguez, 2020). Herein lies one of the key conundrums that is facing all healthcare workers globally but can become a significant challenge for SLTs in SA. As a result of the ever-increasing number of cases that are being admitted into hospitals, there is a significant shortage of PPE in all healthcare facilities. Social distancing is also now a necessity in order to avoid transmission. Considering the importance of social distancing and the lack of PPE, we loop back to the question of whether SLTs should be changing the types of patients who are being regularly referred for dysphagia assessments in this era by implementing a triaging approach to referrals.

Should SLTs be assessing all patients for dysphagia, or do certain patients need to be prioritized based on their medical risk factors and case history? An older study by Langmore et al. (1998) analysed various clinical criteria that could be used as predictive factors for patients who were at risk of developing aspiration (Langmore et al., 1998). This study implies that using medical histories, more at-risk patients can be identified successfully. There have been various online discussion forums (COVID Era and SLP dysphagia practice on Apple Podcasts, 2020) from the United States (US) that also suggest that certain ‘at-risk patient’ protocols are already in place at some healthcare institutions. The concept implies that prioritizing certain patients who are potentially more at risk of developing a dysphagia reduces exposure and potential transmission and requires less PPE. A suggestion for this type of criteria, as mentioned in the same online discussion forum, was that more medically unstable patients who have been referred specifically by a physician for signs and symptoms of a dysphagia, need to be prioritized. Also, patients with a particular condition that is known to cause a dysphagia or patients with a history of dysphagia require more frequent monitoring. When investigating this topic, a gap emerges. There appears to be more literature around what tools are appropriate for the screening and assessment of dysphagic patients (Audag, Goubau, Toussaint, & Reychler, 2019; Sassi, Medeiros, Zilberstein, Jayanthi, & Andrade, 2017) compared to what criteria can be used to identify these patients by other professionals prior to a screening or assessment by an SLT. Perhaps, this is an area that needs to be developed.

For many SLTs in SA, this may require physicians to be trained more specifically on dysphagia so that they are also being more cognisant of who is considered to be at risk and therefore should be referred. Because of the newness of this pandemic, data on the prevalence and description of dysphagia in COVID-19 patients specifically in the SA context would be valuable here for future research trajectories. This is important to inform improved context-specific practices and policies.

This question regarding the role of dysphagia assessments also becomes significant in light of the fact that ear, nose and throat (ENT) doctors have essentially stopped all non-emergent (i.e. everything other than emergent airway cases) endoscopic procedures because of the high risk of transmitting COVID-19 with these procedures (ENT United Kingdom [UK] Guidelines for changes in ENT during COVID-19 pandemic, 2020). So, in that case, what does it indicate about the need for and access to instrumental measures for dysphagia assessments if the use of endoscopic evaluations (i.e. FEES) becomes severely limited? How would this change SLT assessment protocols and guidelines? Another factor to consider is the use of videofluoroscopy for assessments, as the need to disinfect the equipment and the room and the risk of exposing a larger team of people for one assessment become a challenge. The SASLHA guidelines (2020) recommend that such assessments only be conducted when emergent and essential. Again, the need for a set of criteria to identify emergent and essential patients is clear.

This shift in focus regarding how and when assessments should be conducted is interesting considering that SLTs are functioning in a technologically driven world, where instrumental measures have become the norm and are the gold standard assessment tools (Costa, 2010). This COVID-19 era has once again levelled the playing field amongst SLTs from different parts of the world by limiting all SLTs’ access to instrumental measures. This has left the humble and often-overlooked clinical swallow evaluation (CSE) as the primary first-line diagnostic tool. There is a significant body of research from within the dysphagia field that has firmly placed the CSE predominantly as a screening tool because of its lower sensitivity and specificity (Kertscher, Speyer, & Palmieri, 2014; Lim et al., 2001; Tohara, Saitoh, Mays, Kuhlemeier, & Palmer, 2003) as a result of its lack of visual input on the actual swallow anatomy and physiology. That said, this era in which SLTs find themselves now requires a significant shift in thinking from all SLTs regarding what information can actually be obtained from a CSE and how SLTs can use it diagnostically in this acute phase to achieve assessment goals. The study by Langmore et al. (1998) is a good start, as it highlights how SLTs can focus on other clinical data sets of the dysphagia assessment battery when needing to make acute diagnostic decisions at the bedside. Adding other measures such as pulse oximetry and cervical auscultation as an adjunct to the CSE could also assist this decision-making process at the bedside (Bergström, Svensson, & Hartelius, 2014; Zhou, Salle, Daviet, Stuit, & Nguyen, 2011).

There is research advocating for a focus on critical thinking and how SLTs make decisions at the bedside with the physiological information that is readily available. What makes SLTs clinicians rather than merely technicians is this ability to critically evaluate and make immediate diagnostic decisions at the bedside. This process is multifaceted and based on a variety of factors such as patient and environmental factors, clinical experience and exposure, and theoretical understanding (Croskerry, 2009; Plowman & Humbert, 2018; Vose, Kesneck, Sunday, Plowman, & Humbert, 2018). This is another suggested area of focus for SA SLTs, especially now that we are in this era of limited access to instrumental measures.

### The role of the multidisciplinary team

Related to the need to reduce the number of contacts with patients and to limit the use of PPE is how SLTs can possibly draw upon other members of a multidisciplinary team to assist in the assessment and management of dysphagia cases. In SA, a multidisciplinary model appears to be the primary model used in acute care settings. This model involves each member working together as a team to achieve a goal, but generally they function independently (Magnus & Turkington, 2006). However, in this COVID-19 era perhaps a more transdisciplinary model needs to be adopted? A transdisciplinary model involves the integration of multiple perspectives to achieve and solve complex challenges (Van Bewer, 2017). This is a more fluid process as to how professionals interact with the patient and each other, with certain team members picking up the responsibilities of others. Perhaps SLTs need to draw on the assistance of nursing or physiotherapy staff in the initial phases of dysphagia assessments? There are potentially significant ethical and scope-of-practice challenges that would need to be overcome before this option would be viable, but it is something that could be considered and further researched. There have been some studies that have highlighted that a multidisciplinary team approach is possible in dysphagia care, but it requires constant monitoring and open communication (Farneti & Consolmagno, 2007; Logemann, 1994). There is a paucity of evidence that focuses on how specifically a transdisciplinary model for dysphagia assessment and management might work, especially in settings such as the ICU, but there is evidence to justify the need for early joint rehabilitation (Taito et al., 2018).

So, in short, yes, dysphagia services from SLTs are and should always be prioritized as essential services.

However, it is a complex answer, as there are some significant risks associated with practising in dysphagia in this era of COVID-19, and it requires some changes in thinking and practices alike. There are also significant benefits for both the patient and healthcare system from this type of intervention. In summary, SLTs should be doing dysphagia practices but not be practising as business as usual.

### The gaps and more questions

Thinking about how SLTs perform dysphagia practices in the COVID-19 era and after having a basic risk–benefit discussion, there are some significant gaps that need highlighting:

- There is currently little dysphagia prevalence or epidemiological data available for South Africa. If we are to justify why dysphagia services are essential, the necessary statistics are required to assist in this argument. This data was severely limited prior to COVID-19 and is now needed even more.
- The SA context is unique because of its under-resourced healthcare system that serves a significant portion of the population (pre-COVID19) (Coovadia, Jewkes, Barron, Sanders, & Mcintyre, 2009). This pandemic can potentially only complicate this situation and place more strain on the system. The role SLTs can play in potentially alleviating certain pressures specifically through early rehabilitation is yet to be determined and needs to be researched. The role of the SLT within the healthcare team needs to explored, and how each member can potentially change or assist in dysphagia assessments to offset these very real risks also needs to be investigated. The notion of potentially using a transdisciplinary approach is one example.
- Considering the quadruple burden of disease that impacts on the SA patient population, the future remains unclear as to the full impact that COVID19 will have on the population. Speech-language therapists need to keep abreast with the latest developments and be considered as frontline healthcare workers to provide this crucial information. This data needs to be recorded for future guidelines and considerations.
- There is a paucity of global data that reflects what dysphagia practices are occurring in different facilities worldwide. There is some data suggesting that dysphagia practice patterns are varied (Andrews & Pillay, 2017; Pettigrew & O’Toole, 2007), which is already problematic. If SLTs were to change practices to suit the demands of the COVID-19 era, there is currently little to no existing baseline data that we can use as a comparison to support our changes in practices. Baseline efficacy studies are therefore one of the types of studies that are needed going forward.
- Practically, during this era, SLTs need to advocate for their roles in high care settings and with patients who have recently required ventilation. There is currently enough data to support this argument when looking at the importance of early rehabilitation in ICUs and PED. Speech-language therapists also need to advocate for the importance of identifying dysphagia early (in all settings) so as to avoid unnecessary hospital visits or longer hospital stays.
- The types of patients who potentially need to be prioritised and who screens these patients are questions that still need answering. Speech-language therapists need to develop criteria-based tools to identify those patients who are most at risk. Perhaps training of other professionals is also important for this.
- The role of telerehabilitation specifically for dysphagia management needs to be investigated. This is important as it may offset some of the risks that have been identified. The limitation of access in the SA context does need to be considered, but this does not necessarily rule it out as an option entirely.
- The current decision-making processes of SLTs during this COVID-19 era and how this links to the evidence-based practice (EBP) model needs to be investigated. The EBP model includes three arms: clinical knowledge, data from the field and patient/contextual factors (ASHA, 2012). There is a need to explore how SLTs are using their clinical expertise, together with existing dysphagia data and with the current tools at their disposal, to negotiate and conduct dysphagia services in this challenging time across different settings in SA specifically. The point raised earlier on the critical use of the CSE and the decision-making process around this, specifically in the SA context, is something that needs to be potentially relooked at and engaged with.

In light of the discussion brought forward, here are some reflective questions that a SLT could use during the COVID-19 era:

- What are my institutional guidelines and policies currently in place regarding essential services?
- What is my current clinical expertise in response to my patients’ needs and contextual circumstances?
- Is my intervention medically warranted for this patient at this stage (risk versus benefit)?

In conclusion, this is an evolving situation, both in terms of the virus itself, how the patient population is presenting and SLT needs and practices. There are currently more questions than answers, but the hope is that this article has started a much-needed conversation and some thinking about how we as a profession can start to address these ever-emerging gaps and questions during this pandemic.
